# How should dexmedetomidine and clonidine be prescribed in the critical care setting?

**DOI:** 10.5935/0103-507X.20210087

**Published:** 2021

**Authors:** Dan Longrois, Fabrice Petitjeans, Olivier Simonet, Marc de Kock, Marc Belliveau, Cyrille Pichot, Thomas Lieutaud, Marco Ghignone, Luc Quintin

**Affiliations:** 1 Hôpital Bichat-Claude Bernard - Paris, France.; 2 Hôpital d’Instruction des Armées Desgenettes - Lyon, France.; 3 Centre Hospitalier de Wallonie Picarde - Tournai, Belgique.; 4 Hôpital de Saint-Jérôme - Québec, Canada.; 5 Hôpital Louis Pasteur - Dole, France.; 6 Hôpital de Bourg-en-Bresse - Bourg-en-Bresse, France.; 7 JF Kennedy Hospital North Campus - West Palm Beach, Fl, United States

**Keywords:** Critical care, Sedation, General anesthesia, Alpha-2 adrenergic agonists, Clonidine, Dexmedetomidine, Guanfacine

## Abstract

Cardiac, ventilatory and kidney management in the critical care setting has been optimized over the past decades. Cognition and sedation represent one of the last remaning challenges. As conventional sedation is suboptimal and as the sedation evoked by alpha-2 adrenergic agonists (“cooperative” sedation with dexmedetomidine, clonidine or guanfacine) represents a valuable alternative, this manuscript covers three practical topics for which evidence-based medicine is lacking: a) Switching from conventional to cooperative sedation (“switching”): the short answer is the abrupt withdrawal of conventional sedation, immediate implementation of alpha-2 agonist infusion and the use of “rescue sedation” (midazolam bolus[es]) or “breakthrough sedation” (haloperidol bolus[es]) to stabilize cooperative sedation. b) Switching from conventional to cooperative sedation in unstable patients (e.g., refractory *delirium tremens*, septic shock, acute respiratory distress syndrome, etc.): to avoid hypotension and bradycardia evoked by sympathetic deactivation, the short answer is to maintain the stroke volume through volume loading, vasopressors and inotropes. c) To avoid these switches and associated difficulties, alpha-2 agonists should be considered first-line sedatives. The short answer is to administer alpha-2 agonists slowly from admission or endotracheal intubation up to stabilized cooperative sedation. The “take home” message is as follows: a) alpha-2 agonists are jointly sympathetic deactivators and sedative agents; b) sympathetic deactivation implies maintaining the stroke volume and iterative assessment of volemia. Evidence-based medicine should document our propositions.

## INTRODUCTION

Circulatory, ventilatory, renal and metabolic management has progressed over the decades, but cognition and sedation are lagging behind. During this interval, the following reversals have occurred: from no sedation to general anesthesia (GA)/deep conventional sedation,^(^1^)^ to interrupted sedation and back^(^2^)^ to minimal sedation.^(^3^)^ Minimal sedation is possible, given repeated nursing reassurance (“reassurance”) and a provision for deeper sedation.^(^3^,^4^)^

**Figure 1:**
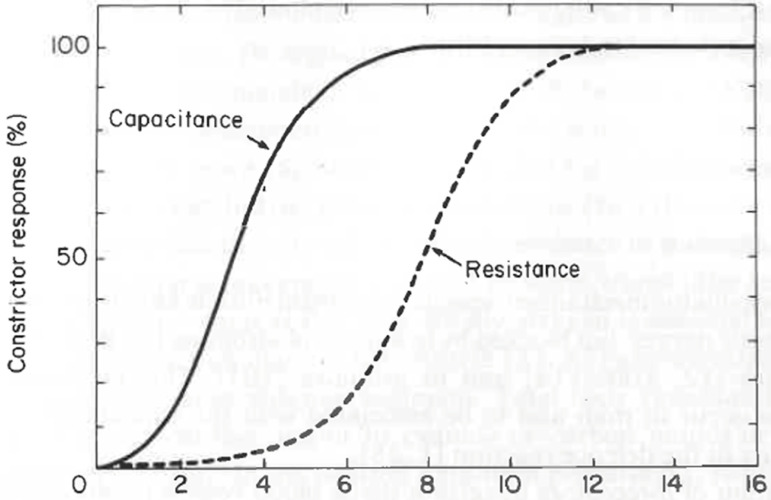
Relationship between smooth muscle activity and the frequency of sympathetic nerve stimuli in capacitance and resistance vessels: frequency-response curve is deduced for resistance (dashed) and capacitance (continuous) in cat skin muscle.

Alpha-2 adrenergic agonists (“alpha-2 agonists”: clonidine, dexmedetomidine, guanfacine) evoke “cooperative”, rousable sedation^#(^5^-^7^)^ and offer an alternative between GA and no sedation. Cooperative sedation reduces the affective-motivational component of pain (indifference to pain, “analgognosia”)^(^8^)^ and evokes indifference to the environment (“ataraxia”) without respiratory depression.^(^9^-^11^)^ The same dose range^(^12^-^14^)^ of alpha-2 agonists that generates cooperative sedation leads to cardiac parasympathetic activation (“cardiac vagal” activation) and attenuation of excessive cardiac and vasomotor sympathetic activity observed in the critical care unit (CCU) back toward baseline (normalization toward baseline: “sympathetic deactivation”; suppressed noradrenaline overflow: “suppressed overflow”). Given the circulatory drawbacks in hypovolemic patients, only niche indications are to be considered (“personalized” medicine), which contradicts the “one size fits all” approach. Circulation is a major concern. In the setting of systolic^(^15^,^16^)^ or diastolic^(^17^)^ failure or cardiogenic pulmonary edema and a low left ventricular (LV) ejection fraction,^(^18^)^ the sympathetic deactivation of capacitance (veins) and resistance vessels (arteries^(^19^,^20^)^) is beneficial. Venous return is reduced, and ejection improves. In the hypovolemia scenario, alpha-2 agonists further reduce venous return (Figure 1^(^21^)^) and stroke volume (SV) and worsen circulatory distress (bradycardia, hypotension, up to cardiac arrest).

Benefits include cognitive^(^6^,^22^-^25^)^ or sleep^(^26^)^ improvements, spontaneous breathing,^(^9^,^11^,^27^)^ improved circulation,^(^15^,^28^)^ kidney function,^(^29^,^30^)^ anti-inflammation^$(^31^-^35^)^ and a reduced CCU stay.^(^36^)^ Outcomes are improved,^(^37^-^45^)^ although the quality of the data suggests waiting for better evidence.

As alpha-2 agonists interfere with the autonomic system *and* cognition (propofol, etc.), problems arise: a) how to switch from conventional sedation to alpha-2 agonists (“switching”), e.g., in agitated or unstable patients, refractory *delirium tremens* (DT), circulatory/ventilatory distress, etc.; and b) how can alpha-2 agonists be prescribed as first-line sedatives de novo upon admission? This manuscript addresses the parasympathetic vs. sympathetic systems, circulation, and ventilation.

Evidence-based medicine is scarce regarding the prescription of alpha-2 agonists. A balanced group of stakeholders with a rigorous approach to the development of consensus guidelines should be convened, which is beyond the reach of our group of lay practitioners: despite its biases, this manuscript is published to help physicians who are not familiar with alpha-2 agonists. Presumably, no formal detailed international guidelines may ever be set up with respect to refractory DT, acute cardioventilatory distress, etc. We reviewed the literature (PubMed search terms: alpha-2 agonist, cooperative sedation, critical care, clonidine, dexmedetomidine, guanfacine). Our clinical practice spanning the period of 1980 - 2020 in several countries (USA, Québec, Belgium, France) is summarized (Table 1). Physiological, pharmacological and clinical matters have been delineated earlier.^(^46^-^48^)^

Effects of lumbar vasoconstrictor fiber stimulation calculated as a percentage of the maximum response for the 2 vascular sections. The curve for the capacitance vessels (veins) is to the left: this curve implies more pronounced effects in this section in the low frequency range compared to those in the resistance vessels (arteries). *One-third of the regional blood volume is expelled at a low frequency stimulation rate, thus increasing venous return*. A fully developed response occurs within 30 - 40 s for both resistance and capacitance vessels. Immediate relaxation occurs after cessation of sympathetic stimulation. In contrast, there is delayed relaxation at high rates of sympathetic stimulation; this delayed relaxation could be of relevance after prolonged administration of high-dose noradrenaline, e.g., in septic shock: prolonged vasomotor sympathetic hyperactivity is associated temporally with poor microcirculation. In turn, prolonged impaired microcirculation is associated with increased mortality. Would normalization of vasomotor sympathetic activity back toward baseline levels be observed and improve microcirculation and outcomes in septic shock?

Source: Prys-Roberts C. Regulation of the circulation. In: Prys-Roberts C, editor. The circulation in anesthesia: applied physiology and pharmacology. Oxford: Blackwell; 1980. p. 179-207. Figure 7.2, Relationship between smooth muscle activity and the frequency of sympathetic nerve stimuli in capacitance and resistance vessels; p. 184.^(^21^)^

**Table 1:** How to prescribe alpha-2 agonist in the critical care setting

A: Switching from conventional to cooperative sedation	
Indications	No "*one size fits all*" approach: positive indications only (cognitive, ventilatory, circulatory, renal, metabolic effects, absence of innate immuno-paralysis, etc.)
Contraindications	Sick sinus syndrome, spontaneous or drug-induced bradycardia, A-V block II/III, uncompensated hypovolemia, liver failure (consider clonidine), renal failure (consider dexmedetomidine unless renal replacement therapy is ongoing or considered)
Drug selection	Dexmedetomidine is easier to use (shorter half-life); clonidine is easier to use through the oral route in nonintubated patients with *delirium tremens*; clonidine or guanfacine p.o. transition from i.v. alpha-2 agonists
	Never use a bolus of alpha-2 agonist: place a "do not bolus" sticker on the iv line of the alpha-2 agonist^(^65^)^
**1 Stable patient**	
Switching from conventional to cooperative sedation	Abrupt withdrawal of conventional sedation followed immediately by i.v. infusion of alpha-2 agonist (dexmedetomidine 1.5µg.kg-1.h-1 or clonidine 2µg.kg-1.h-1 then titration to effect): expect 1 - 3 hours (dex) to 2 - 6 hours (clonidine) before reaching steady-state cooperative sedation
Rescue sedation	The administration of high dose alpha-2 agonist is suggested to reach steady state cooperative sedation as early as possible with minimal rescue sedation. Nevertheless, the dose of alpha-2 agonist has to be lowered to have an effect (-2 < RASS < 0) as early as possible
	Rescue sedation ready at hand in young combative patients: midazolam bolus 3 - 5mg (select the lowest dose; only bolus) to be repeated every 5 - 15 minutes if needed, up to steady-state cooperative sedation
	Titrate dexmedetomidine/clonidine to effect:-2 < RASS < 0; supportive therapy: early physiotherapy, sleep-wake cycle preservation, etc.
	Before nursing, if needed, consider midazolam bolus 3mg with reassurance.
**2 Unstable patient**	
Refractory delirium tremens	
Goal	Supportive therapy as usual (hydration, potassium, magnesium, vitamins, etc.)
	Transient deeper sedation (RASS =-3) during cessation of agitation with alpha-2 agonists combined if needed with neuroleptics
	Then: quiet patient (-2 < RASS < 0): no brisk movement, agitation, hallucination, fine tremor for > 24 hours; as soon as agitation, hallucination, tremor is stopped for > 24 hours, consider tapering drug treatment over 48 - 96 hours
Drug selection	alpha-2 agonist ± neuroleptics (if needed very rarely: ± low-dose benzodiazepine: midazolam 0.5 - 1mg.h-1 ± baclofen)
	Discontinue benzodiazepine, opioid analgesics, etc., immediately upon admission; use benzodiazepines or opioid analgesics only as "rescue" sedation or "rescue" analgesia
	Nonintubated patient: clonidine p.o. 300 - 600µg in small amount of water every 4 hours, then every 6 hours, then every 8 hours, etc., up to 2 - 3µg.kg-1.h-1 for 72 - 96 hours.
	Intubated patient:
	Address volemia iteratively (see below)
	Dexmedetomidine 1.5µg.kg-1.h-1 or clonidine 2µg.kg-1.h-1
	Place a "do not bolus" sticker on the i.v. line^(^65^)^
	When alpha-2 agonists are not sufficient to evoke-2 < RASS < 0 with absence of brisk movement agitation or tremor, supplement with neuroleptics
	a) Hallucinations: haloperidol bolus 5 - 10 mgx4 or 50mg/48mL/24 hours: 2mg.h-1 to be lowered as soon as RASS <-2
	NB: consider haloperidol maximal dose: 30mg.d-1;^(^93^)^ some authors use significantly higher doses
	b) Agitation: loxapine 100mgx4 through nasogastric tube to be lowered to 75mgx4, then 50mgx4, etc., and stopped as soon as possible
	Administer neuroleptic as first-line drug (e.g., haloperidol 5mg i.v. or loxapine 100mg through the nasogastric tube) to avoid abrupt agitation upon withdrawal of conventional sedation and before achieving steady-state cooperative sedation; suppress neuroleptics to make treatment as simple as possible as soon as possible
	NB: monitor QT when administering any neuroleptics
Tracheal extubation	Alpha-2 agonists do not suppress airway reflexes: a) assess clinical status (ventilation, circulation, infection, inflammation, etc.); b) taper neuroleptics first; c) titrate alpha-2 agonists to-2 < RASS < 0, then extubate under continued administration of alpha-2 agonist titrated to-2 < RASS < 0
Tapering alpha-2 agonists	Alpha-2 agonist withdrawal is of rare occurrence: nevertheless, taper i.v. or p.o. alpha-2 agonist over 48 - 96 hours; clonidine p.o. or guanfacine p.o are useful here
Discharge from CCU	Do not discharge the patient early to ward (hallucinations or tremor should be suppressed for > 24 hours): alpha-2 agonists are usually withdrawn on the ward with reintroduction of benzodiazepines, leading to readmission to CCU
Shock/circulatory distress	
Address hypovolemia iteratively	Iterative passive leg raising (PLR, figure 1^(^121^)^) and echocardiography (collapsability of vena cava, etc.; see text) to allow for absence of increase in systemic pressure or in cardiac output following volume loading (e.g., crystalloid bolus 1000mL/70kg patient)
	Volume loading (1000mL bolus/70kg) as long as there is hypovolemia (a pressure or better a cardiac output response to PLR does not necessarily mean that the patient is hypovolemic; figure 1).^(^122^)^ The lung is to be kept “dry”.
	Objective: maintenance of stroke volume,^(^109^)^ diuresis, suppression of mottling, normalization of capillary refill time, lactate, CO_2_ gap, and SsvcO_2_
	"*Start slow, go slow*": dexmedetomidine 0.125µg.kg-1.h-1 for 1 h, then increments of 0.125 to 0.375µg.kg-1.h-1 every hour, up to 1.5µg.kg-1.h-1, according to iterative PLR, echocardiography and circulatory response; rescue sedation only if agitation
	*Or* clonidine 0.125µg.kg-1.h-1 for 1 h, then increments of 0.125 to 0.375µg.kg-1.h-1 every h, up to 2µg.kg-1.h-1, according to iterative PLR, echocardiography and circulatory response, rescue sedation if agitation
	Vasopressors and inotropes according to the usual clinical and echocardiographic indications; no increase in vasopressor or inotrope requirement is observed unless hypovolemia or ventricular failure is not addressed before and during initiation of cooperative sedation
	Antiarrhythmics (amiodarone, verapamil, beta blockers, etc.) are used as indicated if dosage and speed of administration are reduced by 50-75%
Ventilatory distress without circulatory distress	
	Discontinue conventional sedation abruptly; if needed, rescue sedation immediately available to maintain-2 < RASS < 0
	Address all causes of tachypnea/hyperpnea: fever control, agitation, inflammation, lung water, systemic acidosis, poor microcirculation, capnia, and hypoxemia
	Address iteratively volemia and circulatory function: see circulatory distress
	Dexmedetomidine 1.5µg.kg-1.h-1 or clonidine 2µg.kg-1.h-1 then titrated to-2 < RASS < 0
Acute cardioventilatory distress	
	Beyond the goal of the paper aiming at junior staff: stabilize circulation first or ventilation first depending of the clinical situation, and then switch from conventional to cooperative sedation in an itemized manner: "*start slow, go slow*", as described above, in an overtly cautious manner
Antinociception	
	Following steady state cooperative sedation, assess pain: visual analog scale (nonintubated patient) or behavioral pain scale (intubated patient); "medical" patients need little antinociception; "surgical" patients require more antinociception
Nonopioid analgesia	As alpha-2 agonists provide analgognosia and analgesia without respiratory depression, the use of opioids with a respiratory depressant effect appears counterproductive
	a) Ketamine 50 - 100mg.day-1, tramadol 400mg.day-1, nefopam 100mg.day-1/48mL: 2mL.h-1. These dosages are to be reduced by 50 - 75% after 1 - 3 days of full impregnation with an alpha-2 agonist
	NB: in elderly patients administer nefopam 20mg/day for 1 - 2 days, and then increase nefopam if necessary up to 100mg if no cognitive side-effects occur; beware of possible acute urine retention if Foley catheterization is not performed
	NB: tramadol is a weak opioid analgesic acting on µ receptors and is contraindicated if acute kidney insufficiency is present
	To avoid opioid analgesics completely or to stop the administration of tramadol-nefopam early in elderly patients, consider
	b) Amitryptyline (Laroxyl®) 25mg i.v.x4 or lidocaine 0.5mg/kg/h (loading dose: 1mg. kg-1.h-1) or ketamine (0.25mg kg-1.h-1) infusion
	c) Or pregabaline (Lyrica®) 150 - 600mg/day: start with 25mgx2 through n/g (Day 0), then 50 x 2 (Day 2), 75x2 (Day 3), etc.; in the case of pancreatitis or CCU neuromyopathy, consider 150 x 2 up to a total daily dose of 600mg
	c) Gabapentine (Neurontin® 100 - 900mg/day) or carbamazepine (Tegretol® 200 - 400mg/day)
Rescue opioids	Only if needed, after pain assessment, rescue opioid analgesics to be reintroduced sparingly aiming for early spontaneous ventilation, intestinal motility, absence of hyperalgesia
B: De novo cooperative sedation	
Indications	No "one size fits all" approach: positive indications only (cognitive, ventilatory, circulatory, renal, metabolic effects; absence of innate immune paralysis, etc.)
Contraindications	Sick sinus syndrome, bradycardia (spontaneous or drug-induced), A-V block II/III, uncompensated hypovolemia, liver failure (consider clonidine), renal failure (consider dexmedetomidine unless renal replacement therapy is ongoing or considered)
Drug selection	Dexmedetomidine is easier to use (shorter half-life); clonidine is easier to use when the oral route is possible (nonintubated patients with *delirium tremens*); clonidine or guanfacine transition from i.v. alpha-2 agonists to no therapy
	Place a "do not bolus" sticker on the i.v. line: never use a bolus of alpha-2 agonist
Circulatory distress	
	Address volemia and circulation iteratively: A
	Start slow and go slow to administer alpha-2 agonist: A
	Have rescue and breakthrough sedation immediately available: A
	Address volemia before general anesthesia, endotracheal intubation and positive-pressure ventilation+PEEP: consider volume (1000mL/70kg patient)(163)
	Consider very high O2 flow or noninvasive ventilation: oxygenation and suppressed work of breathing (patient-self induced lung injury) prior to intubation
	Dexmedetomidine 1.5µg.kg-1.h-1 or clonidine 2µg.kg-1.h-1 then titrated to-2 < RASS < 0, immediately following noninvasive ventilation or invasive ventilation
Antinociception	
	Assess pain: visual analog scale in nonintubated patient or behavioral pain scale in intubated patient
	Drugs: priority to nonopioid analgesics; use rescue opioid analgesics only; table A

AV - atrioventricular; RASS - Richmond Agitation Sedation Scale; CCU - critical care unit; CO_2_ - carbon dioxide; SsvcO_2_ - superior vena cava oxygen saturation; PEEP - positive end-expiratory pressure.

## SWITCHING FROM CONVENTIONAL SEDATION TO COOPERATIVE SEDATION

Conventional sedation combines benzodiazepine or short-acting general anesthetics with opioid analgesics. Muscle relaxants are mainly used in the setting of acute respiratory distress syndrome (ARDS), traumatic brain injury,^(^49^)^ etc. Nevertheless, a) emergence *delirium* is encountered following deep sedation. However, is this *delirium* related to the pathology itself, the CCU environment, or conventional sedation? Moreover, b) deep sedation, bordering GA (1), is used in clinical practice for ARDS or increased intracranial pressure^(^49^)^ without evidence.^(^50^)^ Indeed, mortality is reduced using controlled mechanical ventilation (CMV), paralysis and proning.^(^51^,^52^)^ Nevertheless, a comparison of deep sedation + CMV + paralysis *versus* adequate spontaneous breathing^(^50^,^53^-^56^)^ is missing.^(^50^)^ Therefore, these advances^(^51^,^52^)^ fall short methodologically, given a) the absence of a control group under adequate spontaneous breathing^(^50^)^ and b) the tendency to shorten^(^57^,^58^)^ GA + CMV + paralysis. An established practice^(^51^,^52^)^ without strong evidence^(^50^)^ faces unorthodox practice^(^59^,^60^)^ or recent proof of concept.^(^53^,^61^)^

As most groups use cooperative sedation after conventional sedation, i.e., only when the patient is recovering and ready for tracheal extubation (“extubation”), switching from conventional to cooperative sedation is examined first.

### Contraindications

Dexmedetomidine and clonidine are sympathetic inhibitors in healthy resting supine volunteers. In the CCU, given the increased sympathetic activity, they normalize sympathetic hyperactivity back toward baseline, i.e., sympathetic deactivators, with the following contraindications:

- Hypovolemia: See below.
- Bradycardia (spontaneous or drug-induced, e.g., by beta-blockers^&^), sick sinus syndrome, atrioventricular block II or III without a pacemaker.
- Liver failure (Child-Pugh C): Clonidine and dexmedetomidine are excreted through the kidney and liver, respectively. Moreover, clonidine and dexmedetomidine are useful in the scenarios of liver and kidney failure, respectively. Nevertheless, a) clonidine can be administered in the setting of acute renal failure if renal replacement therapy (RRT) is used, and b) dexmedetomidine can be used in the setting of liver cirrhosis.^(^62^)^

### Clonidine versus dexmedetomidine

The higher alpha-2/alpha-1 receptor selectivity of dexmedetomidine is of *no* clinical relevance but is only an *in vitro* finding.^(^63^)^ Rather, dexmedetomidine, also available p.o.,^(^64^)^ is implemented more easily by nurses than clonidine is (Simonet and de Kock, personal communication). In contrast, clonidine p.o. allows for convenient oral administration (nonintubated patient with DT), transitioning alpha-2 agonists from i.v. dexmedetomidine to p.o. clonidine to avoid alpha-2 agonist withdrawal, etc. Sedation is achieved within 30 - 60 minutes in healthy volunteers after clonidine 300µg p.o.^(^5^,^12^)^

### Progressive versus abrupt switching

**Abrupt withdrawal:** Abrupt withdrawal of conventional sedation to achieve-2 < Richmond Agitation Sedation Scale (RASS) < +1 occurs immediately before initiation of dexmedetomidine infusion^(^23^)^ (0.8µg.kg-1.h-1; loading bolus =1µg.kg-1 if necessary; infusion range: 0.15 - 1.5µg.kg-1.h-1^(^22^)^). Rescue sedation is used to achieve-2 < RASS < +1^(^23^)^ using either a) fentanyl infusion, followed by a propofol bolus (25 - 50mg)^(^22^)^ or b) midazolam (0.01 - 0.05mg.kg-1 per 10-minute intervals to a total of 4mg/8h) and fentanyl.^(^23^)^

**Figure 2:**
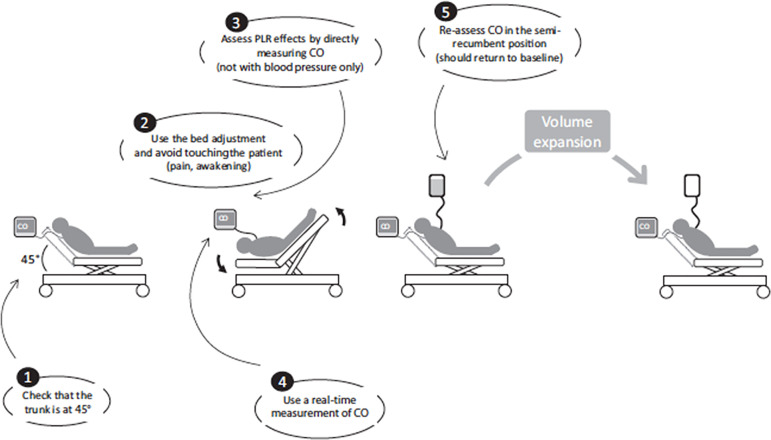
Iterative passive leg raising (PLR) to address hypovolemia before administration of an alpha-2 agonist in patients presenting with circulatory instability.

**Progressive switching:** Withdrawal of conventional sedation is set over 2 hours. Meanwhile, the introduction of cooperative sedation was implemented over the same time interval (dexmedetomidine 0.4µg.kg-1.h-1, increased progressively to effect; [Figure 2^(^65^)^]; a *“*do not bolus” sticker was placed on the electric syringe and infusion line^(^65^)^). A “ceiling” effect is reported with dexmedetomidine >1.5µg.kg-1.h-1.^(^7^)^ High-dose clonidine is 2µg.kg-1.h-1;^(^66^)^ there is no reported ceiling effect. During the switch, before achieving steady-state cooperative sedation, rescue sedation is administered with boluses of midazolam (1mg) or propofol (25mg) to be repeated if necessary.^(^65^)^ Progressive switching requires experienced intensivists and critical care nurses.^(^65^)^ The drawbacks of progressive switching or of combined administration of dexmedetomidine with conventional sedation are as follows:

- Progressive switching and circulation: Simultaneous administration of conventional sedation and cooperative sedation combines the sympathetic deactivation evoked by alpha-2 agonists,^(^67^)^ the sympathetic inhibition evoked by propofol^(^68^)^ and the parasympathetic activation evoked by opioids; this leads to a low heart rate (HR), blood pressure (BP) and cardiac output (CO).^(^69^)^ If the patient under alpha-2 agonist infusion becomes agitated or restless, he may inappropriately receive an additional bolus of high-dose propofol (50 - 100mg) or a bolus of clonidine/dexmedetomidine. Consequently, severe bradycardia and hypotension may occur. To avoid such side effects, we used abrupt withdrawal. Abrupt withdrawal is performed during the day shift only, starting in the early morning^£^. The prescription specifies the target (-2 < RASS < 0), the range of dose of dexmedetomidine (≤ 1.5µg.kg-1.h-1), the rescue *versus* breakthrough sedation (rescue: midazolam 3 - 5mg repeated every 5 - 10 minutes up to-2 < RASS < 0; no propofol or thiopentone bolus except for brisk agitation and a “stat order” with the intensivist by the bedside; breakthrough: haloperidol bolus 5-10mg), and the supplementation (neuroleptics: see refractory DT).
- Combined cooperative and conventional sedation: An additive effect between opioids and alpha-2 agonists was delineated, with bradycardia and lowered CO.^(^69^)^ Indeed, administration of clonidine pre- and postoperatively to patients administered the *same* dose of conventional GA led to bradycardia, hypotension and cardiac arrest^(^70^)^ without sequelae.^(^71^)^ In the CCU, dexmedetomidine (1 - 1.5µg.kg-1.h-1, up to-2 < RASS < +1) led to no change in mortality (SPICE III).^(^72^)^ Greater bradycardia and hypotension were observed with combined cooperative and conventional sedation than with conventional sedation alone.^(^72^)^ However, deep sedation was used in ≈60% of conventional sedation patients (Day 1), while ≈75% of dexmedetomidine patients received propofol, midazolam or both.^(^72^,^73^)^ Therefore, any difference is obscured, and this trial^(^72^)^ is useless.^(^73^)^ A *post hoc* analysis comparing dexmedetomidine alone to conventional sedation alone is needed^(^73^,^74^)^ to reassess the outcome and make this large series^(^72^)^ useful.

Passive leg raising combines bed adjustment with monitoring of the cardiac output or blood pressure response. Passive leg raising separates volume-responsive versus nonresponsive patients: *volume-responsive patients are not necessarily in a hypovolemic state and do not necessarily need additional volume*. If needed, the patients not responding to PLR requires a vasopressor and/or inotropes.

CO - cardiac output.

Modified from: Monnet X, Teboul JL. Passive leg raising. Intensive Care Med. 2008;34(4):659-63; Monnet X, Teboul JL. Passive leg raising: five rules, not a drop of fluid! Crit Care. 2015;19(1):18. Figure 1, The best method for passive leg raising, indicating the five rules to be followed; p. 2.^(^121^,^122^)^ [creative commons attribution license].

In summary, mixing conventional sedation with cooperative sedation in the operating room^(^69^,^70^,^75^)^ or CCU^(^72^)^ leads to severe circulatory side effects.

### Switching in the setting of preoperative, intraoperative and postoperative administration of alpha-2 agonists

Two situations may be considered:

Intraoperative opioid-free anesthesia was administered:^(^76^)^ the alpha-2 agonist was administered (see below).

Intraoperative conventional GA has been administered: given premedication with an alpha-2 agonist^(^13^)^ or intraoperative administration of an alpha-2 agonist,^(^76^)^ if intraoperative opioids and general anesthetic administration have been reduced by 50-75%,^(^13^,^77^-^79^)^ then cooperative sedation is administered when reaching the CCU if the expected CCU length of stay is > 2 days. The dose of alpha-2 agonists (e.g., clonidine 900µg pre- and intraoperatively for aortic surgery;^(^80^)^ 4µg.kg-1/15 minutes during the induction of anesthesia for liver transplant^(^28^)^) is usually sufficient to cover the first postoperative day, with provision for opioid-free analgo-sedation and nicardipine (0.5mg to be repeated if needed). Technology addresses volume (pleth variability index, passive leg raising [PLR], echocardiography) or perfusion (ST monitoring, cerebral oxygenation). If, after volume adjustment, the perfusion pressure is a concern, adjuncts (very low dose noradrenaline 0.01 - 0.03µg.kg-1.min-1,^(^81^)^ compression stockings, lower limb elevation) are used to counteract sympathetic deactivation. An additive effect between the incoming alpha-2 agonist and the opioid^(^69^)^ should be eliminated. ii) If conventional GA has been administered, low-dose alpha-2 agonists will be introduced slowly (e.g., dexmedetomidine 0.4 - 0.7µg.kg-1.h-1) to effect.

### Titration to effect

The required RASS (-2 < RASS < 0) deserves comments:

- We do not use-2 < RASS < +1 as others do:^(^23^)^ Stringent absence of restlessness without any regular, repeated, brisk limb movements is required. In our practice, a patient presenting with the rare occurrence of brisk limb movements may present sudden agitation, assume an erect position and withdraw catheters and tubing in the middle of the night. First, to achieve stringent restlessness, alpha-2 agonists are administered up to the ceiling^(^7^)^ effect (dexmedetomidine 1.5µg.kg-1.h-1 for ≥ 3 hours; clonidine: 2µg.kg-1.h-1 for ≥ 6 hours). Second, if needed after this interval, neuroleptics are administered. This avoids the cognitive side effects of benzodiazepines (below: refractory DT). Midazolam is used only as rescue sedation during the switch, e.g., to facilitate nursing.
- Elderly patients appear less sensitive to the sedation evoked by alpha-2 agonists than young, muscular, combative, and addicted^(^82^)^ patients. Sleep is induced by carotid massage in young individuals,^(^83^)^ i.e., possibly via cholinergic activation. In contrast, aging and a loss of forebrain cholinergic receptors are compatible with reduced sedative effects in elderly patients. Adequate sedation in elderly patients requires either very high doses of alpha-2 agonists (clonidine 4µg.kg-1.h-1; dexmedetomidine 2.5µg.kg-1.h-1) or low-dose neuroleptics added to high-dose alpha-2 agonists (clonidine 2µg.kg-1.h-1; dexmedetomidine 1.5µg.kg-1.h-1).

### Antinociception

Once steady-state cooperative sedation is achieved, antinociception is considered. Patients presenting with medical conditions require little antinociception^(^7^)^ but only analgognosia^(^8^)^ and ataraxia, addressed by the alpha-2 agonist. In contrast, surgical patients present higher antinociceptive requirements.^(^7^)^ After assessment of the Visual Analog Scale (VAS) or Behavioral Pain Scale (BPS) score, opioids^(^22^,^23^)^ (fentanyl 0.5 - 1µg.kg-1 every 15 minutes) or nonopioid analgesics can be selected. However, a) alpha-2 agonists evoke analgognosia^(^8^)^ and preserve respiratory genesis.^(^9^-^11^)^ Nonopioid analgesics provide antinociception and preserve spontaneous breathing. Therefore, our protocol is as follows:

- Nefopam (100mg.d-1), low-dose ketamine (50mg.d-1) and tramadol (“weak” opioid: 400mg.d-1). These doses are reduced by 50 - 75% after 24 - 72 hours. This may be a consequence of accumulation or the indifference to pain evoked by the alpha-2 agonist following steady-state cooperative sedation.
- or lidocaine 0.5mg.kg-1.h-1 infusion (loading dose: 1mg.kg-1.h-1) or ketamine (0.25mgkg-1.h-1) infusion or gabapentin (Neurontin®, 100 - 900mg.day-1 [d-1]) or pregabalin (Lyrica®, 150 - 600mg.d-1) or carbamazepine (Tegretol®, 200 - 400mg.d-1) or amitriptyline (Laroxyl®, 12.5 - 25mg i.v. especially in the postoperative setting). Low-dose opioids are employed as rescue analgesics, if needed.

### Overdose of alpha-2 agonists

In the setting of ambulatory cardiology, very high-dose alpha-2 agonists lead to resistant hypertension (clonidine 5400 - 6000µg.d-1).^(^84^,^85^)^ High-dose dexmedetomidine (4µg.kg-1.h-1 for several hours) leads to hypertension and low HR (60 - 70 beats per min in a 2-year-old child) without sequelae upon reduced dexmedetomidine administration.^(^86^)^ Intentional or accidental overdose leads to minimal side effects: sedation, hypotension, bradycardia, and no respiratory depression^.(^87^-^89^)^ Naloxone does not revert sedation.^(^89^)^ This margin of safety should not allow one to forget to address contraindications (see above).

## SWITCHING IN UNSTABLE PATIENTS

### Refractory delirium tremens

Alpha-2 agonists have been used in the setting of refractory DT to supplement conventional sedation.^(^90^-^92^)^ Recently,^(^93^)^ low-dose dexmedetomidine (0.7µg.kg-1.h-1) was successfully supplemented with haloperidol in nonintubated patients (goal: RASS = 0; maximum haloperidol dose: 30mg.day-1 [d-1]). Dexmedetomidine achieves ≈93% satisfactory sedation levels (haloperidol ≈60%) and halves the CCU stay.^(^93^)^

The rationale for using alpha-2 agonists as first-line agents up to the “ceiling” effect,^(^7^)^ with neuroleptics as second-line agents, on an *ad hoc* basis, is as follows:

- DT involves hyperactivity or hypoactivity of several central pathways (noradrenaline via alpha-2 receptors, dopamine, glutamate *versus* GABA). Thus, a combination of drugs manages a complex neurochemical pattern.
- Alpha-2 agonists lower the baseline activity of noradrenergic neurons but increase their reactivity^(^94^)^ (lowered “tonic” background activity, i.e., suppressed overflow *versus* increased “phasic” activity). The signal-to-noise ratio^(^95^)^ and the gain of the central noradrenergic dorsal system increase.^(^96^)^ Clinically, the patient is quiet and sedated (stage 2 sleep;^(^26^,^97^)^-2 ≤ RASS ≤ 0) but “fairly alert”^(^5^)^ or cognitively improved^(^24^)^ upon a stimulus.
- The muscular tremor is abated,^(^98^,^99^)^ and the temperature^(^100^)^ and oxygen consumption (VO_2_)^(^101^-^103^)^ are lowered.

When high-dose alpha-2 agonists (dexmedetomidine 1.5µg.kg-1.h-1; clonidine 2µg.kg-1.h-1) are insufficient to achieve-2 ≤ RASS ≤ 0 (stringent absence of restlessness) without tremor, neuroleptics are employed as second-line agents. When hallucinations were prominent, haloperidol (bolus: 5mg four times per day: 5mg x 4 i.v.; or infusion: 50mg/48mL/24h: 2mL.h-1, to be lowered as soon as possible) is administered. In contrast, when agitation was prominent, loxapine (100mg x 4 p.o. or via the nasogastric tube) is selected. Neuroleptics, then alpha-2 agonists, are tapered as soon as the absence of restlessness without tremor is ascertained for 24 hours.

Refractory DT patients with Gayet-Wernicke disease required clonidine 4 µg.kg-1.h-1+loxapine 400mgx4 to achieve-2 ≤ RASS ≤ 0 and the absence of tremor. To supplement a combination of high-dose alpha-2 agonist + neuroleptic (dexmedetomidine 1.5µg.kg-1.h-1 + haloperidol up to 50mg.d-1; clonidine 2µg.kg-1.h-1 + loxapine 100mgx4) and to avoid the administration of higher doses of alpha-2 agonists + neuroleptics, baclofen (50 - 150mg according to kidney function)^(^104^)^ or low-dose midazolam (0.5mg.h-1) may be considered.

Refractory DT in nonintubated patients^(^93^)^ is an issue. Do they require GA + intubation? These patients present short bouts without agitation or restlessness. Thus, young, combative, addicted patients are able to swallow clonidine (p.o. 7.5 - 10µg.kg-1.h-1; pills crunched or vials in a minimal amount of water) and achieve quietness within 30 - 60 minutes. A similar regimen may be used to transition from i.v. dexmedetomidine to oral clonidine (300µg every 6 hours, then 9 hours, then 12 hours, etc.),^(^105^)^ up to discontinuation.^(^105^)^ In this respect, guanfacine (Estulic®; half-life: 10 - 30 hours or extended-release guanfacine: Intuniv®) may be considered to initiate oral therapy or to transition from i.v. dexmedetomidine to an oral alpha-2 agonist.

### Circulatory distress

Given the contraindications (see above), the administration of alpha-2 agonists is inadvisable in the setting of uncontrolled hemorrhage, septic or cardiogenic shock, etc. Indeed, for a short period of time, sympathetic activation is a lifesaver in regards to control of the pathology, and exogenous vasopressors and/or inotropes are required to maintain left ventricular perfusion pressure and/or contractility, in addition to endogenous sympathetic nervous activation. In contrast, AFTER controls acute cardioventilatory distress, and alpha-2 agonists deactivate the prolonged sympathetic hyperactivity observed in the CCU. After circulatory optimization, normalized sympathetic hyperactivity toward baseline may benefit metabolic syndrome, immunoparalysis, etc., e.g., in the following settings: circulatory failure following cardiac surgery^(^106^,^107^)^ or low ejection fraction in the medical setting;^(^18^)^ Sepsis;^(^39^)^ mild,^(^108^)^ severe^(^109^,^110^)^ or refractory^(^111^)^ septic shock; or unclamping of a liver graft,^(^28^)^ with lowered noradrenaline requirements.

Sympathetic hyperactivity is normalized back toward baseline by alpha-2 agonists; background activity is lowered. A reduced noradrenaline overflow in the synaptic cleft leads to reactivation of alpha-1 receptors: desensitized receptors return to baseline activity (“upregulation”;^(^108^-^110^,^112^-^114^)^ “denervation hypersensitivity”^(^112^,^115^)^). Increased pressor responsiveness to noradrenaline toward baseline follows.^(^113^,^114^)^ Presumably, improved microcirculation^(^28^,^116^)^ extends this upregulation to the peripheral capillaries. Progressive sympathetic vasomotor deactivation in capacitance vessels (veins)^(^21^,^117^)^ is combined with volume loading, which maintains venous return.^(^109^)^ Increased LV compliance^(^17^)^ and vasomotor sympathetic deactivation in resistance vessels (arteries)^(^15^,^16^,^118^)^ and lowered LV impedance^(^19^,^20^)^ maintain the SV. Any hypotension, bradycardia or supraventricular arrhythmia relates to lowered venous return, coronary perfusion pressure or compliance.

**Figure 3:**
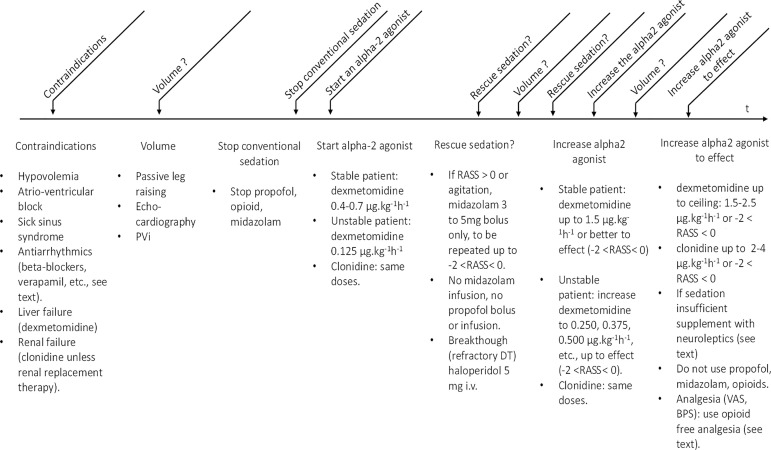
“Start slow, go slow” administration of alpha-2 agonists in patients presenting with circulatory instability.

Drugs combining sedation and sympathetic deactivation modify the circulation and require the following:

- Abrupt withdrawal of conventional sedation with rescue sedation as needed, up to steady-state cooperative sedation. However, in the conditions of low flow or pressure, the requirements for rescue, conventional or cooperative sedation are usually minimal.
- No hypovolemia: Following alpha-2 agonist administration, SV maintenance is required:^(^109^)^ further volume loading will not evoke any further increase in CO or BP following PLR. To achieve SV maintenance, different protocols were used: 1500mL of fluid;^(^109^)^ 10mL.kg-1;^(^65^)^ and a combination of the following:
  - First, after each bolus (1000mL/70kg) or each increment of alpha-2 agonist, absence of or minimal collapsibility of the vena cava^(^119^,^120^)^ and/or increase in CO or BP following adequate PLR (Figure 3^(^121^,^122^)^): PLR separates the volume-responsive versus nonresponsive patients: the volume-responsive patients are not necessarily in a hypovolemic state and do not necessarily need additional volume. Volume is minimized to prevent increased lung water.^(^122^,^123^)^ Nevertheless, following dexmedetomidine, 5 out of 20 patients with septic shock switched from preload independence to preload dependence.^(^124^)^ This may evoke hypotension within the first 3 hours of administration^(^125^)^ and suggests iterative circulatory optimization.
  - Second, the adequacy of CO and microcirculation are addressed: diuresis, capillary refill, mottling, lactate,^(^28^,^116^,^126^)^ O_2_ arteriovenous difference^(^127^)^ or superior vena cava oxygen saturation (SsvcO_2_), carbon dioxide (CO_2_) gap.
- Slow administration of a low-dose alpha-2 agonist (dexmedetomidine 0.125µg.kg-1.h-1 i.v. increased incrementally to 1.5µg.kg-1.h-1 over 3 - 12 hours). We propose this overtly cautious approach and termed it *“*start slow, go slow”, borrowed from the administration of beta-blockers in heart failure^(^128^)^ (Figure 3). No alpha-2 agonist bolus is ever administered. Indeed, a high alpha-2 agonist concentration (bolus) will first stimulate vascular alpha-1 receptors, leading to paradoxical hypertension. After dilution of the bolus, brain stem alpha-2 receptors are stimulated, deactivating vasomotor sympathetic hyperactivity, enlarging venous capacitance, and reducing venous return^(^124^)^ (Figure 1).^(^21^)^

In summary, bolus alpha-2 agonist administration with simultaneous conventional sedation administration or without the iterative assessment of volemia leads to severe bradycardia and hypotension.

Antiarrhythmics are relative contraindications: for simplicity, the beta blockers verapamil and amiodarone are to be withdrawn over 24 - 72 hours. Evidently, if supraventricular arrhythmia occurs during initiation of the alpha-2 agonist, antiarrhythmics should be administered, with the dose and speed of administration half and then titrated to effect. The perfusion pressure and electrolytes are restored. Administration of an alpha-2 agonist is appropriate only after optimization of volemia (Figure 3). Alpha-2 agonist administration is initiated immediately after the withdrawal of conventional sedation. Usually, low flow or septic confusion makes the transition uneventful. In young, combative patients, rescue sedation (midazolam 3 to 5mg repeated every 5 to 10 minutes) maintains-2 < RASS < 0. In the setting of refractory *delirium tremens*, neuroleptic administration *before* the withdrawal of conventional sedation will avoid breakthrough (e.g., loxapine 100mg p.o. or through a nasogastric tube, or haloperidol 50mg/48mL, 2mL.h-1; breakthrough sedation: haloperidol: 5 - 10mg bolus). In unstable, hypovolemic, hypotensive, hypoxic patients, the administration of alpha-2 agonists was increased from 0.125 to 1.5mg.kg-1.h-1 over 3 to 12 hours. *Iterative* passive leg raising and echocardiography allow for incrementing the administration of the alpha-2 agonist: *venous return should be appropriate, and the stroke volume should be maintained throughout the administration of the alpha-2 agonist*.^(^109^)^ Opioid-free analgesia in the setting of cooperative sedation avoids respiratory and cognitive depression. Opioids are only used as rescue analgesia.

PVi - plethysmography variability index; RASS - Richmond agitation and sedation scale; VAS - Visual Analog Scale; BPS - Behavioral Pain Scale; PVi - plethysmography variability index; DT - *delirium tremens*.

### Ventilatory distress

Established practice^(^51^,^52^)^ presents shortcomings.^(^50^)^ Alternative practices^(^53^,^59^,^61^)^ are in their infancy.

Switching from conventional to cooperative sedation in the setting of hypovolemia and vasopressor administration addresses only one circulatory issue. In the setting of ventilatory distress, circulatory distress is intermingled with ventilatory distress:^(^129^)^ respiratory arrest usually occurs before cardiac arrest and requires addressing ventilatory distress upfront; positive pressure ventilation with positive end-expiratory pressure (PEEP) imposed on hypovolemia worsens circulatory distress.

### Switching in a stable patient

Switching is considered for a patient who has recovered from acute respiratory distress, i.e., before switching to spontaneous breathing. Conventional sedation is abruptly withdrawn. Dexmedetomidine was introduced (up to 1.5µg.kg-1.h-1 incrementally over 2 - 3 hours or, better, to effect:-2 ≤ RASS ≤ 0; see “circulatory distress”). Rescue sedation is administered if needed. Muscle relaxants are withdrawn immediately before steady-state cooperative sedation is established, with reassurance. Spontaneous breathing is established as soon as^(^130^)^ the factors evoking increased inspiratory activity are controlled (“respiratory drive”, tachypnea and hyperpnea): fever control,^(^131^-^133^)^ agitation,^(^103^,^134^)^ inflammation,^(^135^,^136^)^ lung water,^(^123^)^ systemic acidosis^(^136^-^138^)^ and microcirculation, mild permissive hypercapnia (40 < PaCO_2_ ≤ 50mmHg),^(^10^,^61^)^ and upright positioning.^(^139^)^ This was delineated^(^53^,^54^,^56^,^61^,^137^)^ in table 1 in the reference.^(^55^)^ The respiratory drive is not to be suppressed pharmacologically with GA, opioids or muscle relaxants but is used physiologically. The respiratory generator is unaffected by alpha-2 agonists.^(^11^)^ In contrast, general anesthetics, benzodiazepines, or opioids suppress the activity of the respiratory generator. Each of the factors enumerated above generates tachypnea and hyperpnea and is addressed separately, a differentiation impossible under GA. The physiological control of increased inspiratory activity leads to the absence of patient self-inflicted lung injury (P-SILI).^(^140^,^141^)^ Then, the patient handles *only* one last factor of increased inspiratory activity, i.e., only hypoxemia under low PS-high PEEP^(^53^-^56^,^142^)^ and cooperative sedation. Low driving pressure,^(^143^-^147^)^ plateau pressure, minimal activity of inspiratory accessory muscles and no sternal notch retraction were observed.

When acceptable, given-2 ≤ RASS ≤ 0, tracheal extubation is achieved without withdrawal of alpha-2 agonists: as alpha-2 agonists do not depress airway reflexes even when very high doses are used,^(^84^,^148^)^ the issue is not the dose of alpha-2 agonist that is administered but the degree of alertness versus deep sedation to allow for extubation. Continuous NIV+PEEP is conducted under continued alpha-2 agonist administration titrated to-2 ≤ RASS ≤ 0 up to weaning.

In summary, the management becomes analytical: administration of an alpha-2 agonist allows one to separate the physiological *versus* pharmacological factors involved in the management of ventilatory distress (increased inspiratory activity *versus* depressed or preserved respiratory generator; ataraxia^(^8^,^149^)^ *versus* deep sedation).

### Switching in an unstable patient

Switching in a patient presenting with acute cardioventilatory distress under conventional sedation in the CCU involves prioritizing between simultaneous issues beyond the scope of this manuscript: stabilized circulation (see above), stabilized ventilatory distress (very high oxygen flow, NIV *versus* controlled mandatory ventilation^(^150^)^), and switching from conventional to cooperative sedation (see above).

## INITIATION OF DE NOVO COOPERATIVE SEDATION

Upfront administration of cooperative sedation is simpler than switching: spontaneous breathing^(^9^-^11^)^ and cognition^(^24^,^25^)^ are not deteriorated by first-line alpha-2 agonists.

### Isolated ventilatory distress

Dexmedetomidine (infusion: 0.7µg.kg-1.h-1) addresses agitation in patients treated with NIV presenting with postoperative ventilatory failure.^(^151^)^ The RASS normalizes itself to-3 < RASS < 0 over 3 hours.^(^151^)^ Simultaneously, the respiratory rate (RR), PaO_2_/FiO_2_ (P/F), HR, and systolic BP normalize. The patient is discharged without intubation.^(^151^)^ Accordingly, dexmedetomidine 0.7µg.kg-1.h-1 eases the adaptation to NIV in the setting of chest trauma.^(^152^)^ These reports need replication.

A similar positive outcome was observed in the setting of severe bronchospasm (dexmedetomidine: 0.25 - 0.8µg.kg-1.h-1)^(^153^-^155^)^ or status asthmaticus (dexmedetomidine: 0.2 - 0.7µg.kg-1.h-1).^(^156^)^ Clonidine (4µg.kg-1 p.o.) achieves the same effect.^(^157^)^ The dose of alpha-2 agonists should be increased, e.g., up to a high dose (dexmedetomidine 1.5µg.kg-1.h-1, or clonidine 2µg.kg-1.h-1) and titrated to effect: stringent ataraxia is requested when psychogenic stimuli are presented.

### Acute cardioventilatory distress

Septic shock^(^158^)^ or early diffuse ARDS^(^150^)^ are beyond the scope of this section. SARS-CoV-2-ARDS (COVID-ARDS) is an inflammatory disease leading to a high respiratory drive and an inflammatory vascular disease of the pulmonary capillaries. Low or medium PEEP is required, with tight control of temperature, agitation and inflammation

Noninvasive ventilation (low PS,^(^143^,^159^-^161^)^ high FiO_2_, high PEEP) or very high O_2_ flow allows one to buy time, expedite preoxygenation^(^162^)^ and minimize the work of breathing. Simultaneously, volume loading (e.g., 1000mL bolus before endotracheal intubation: “intubation”) prevents the circulatory collapse observed immediately after intubation + positive pressure ventilation + PEEP in hypovolemic patients.^(^163^)^

If NIV partitions the patients in need of CMV *versus* NIV,^(^140^)^ over 30 - 60 minutes, alpha-2 agonist infusion may be started before setting up NIV or during NIV. Conversely, alpha-2 agonists are infused immediately after intubation. Rescue or breakthrough sedation is used up to stable cooperative sedation.

The dose of dexmedetomidine is a function of the circulation (see above: 0.125µg.kg-1.h-1 incrementally up to 1.5µg.kg-1.h-1,-2 ≤ RASS ≤ 0 over 3 - 12 hours: start slow, go slow). As an extended CCU stay is likely, immediate stable cooperative sedation is not warranted. First, stabilization of the circulation should be achieved (volume vs. vasopressors when the diastolic pressure is low^(^164^)^). Second, iterative rescue sedation allows one to stabilize incremental cooperative sedation. Finally, P-SILI and hypoxemia are addressed (Table 1^(^55^)^).

Two issues deserve comment:

- Tolerance to the sedative effects of alpha-2 agonists develops over weeks^(^148^)^ or days. In addition, septic confusion or low-flow obtundation improved over time. Therefore, the sedation achieved with alpha-2 agonists may become insufficient. Higher doses of alpha-2 agonists may be used. Conversely, supplementation with neuroleptics (see above) achieves-2 < RASS < 0.
- Muscle relaxation suppresses P-SILI and patient-to-ventilator dyssynchrony^(^165^)^ for 12-48 hours.^(^51^,^57^,^58^)^ Should first-line alpha-2 agonists administered to the ceiling effect be supplemented under muscle relaxation? Awareness will be minimized by iterative clinical examination, electroencephalography (BIS), titration of alpha-2 agonists to effect, reassurance and additional neuroleptics.

## FINAL CONSIDERATIONS

In the critical care unit, alpha-2 agonists present intrinsically intertwined^(^13^)^ therapeutic effects and side effects, i.e., cooperative sedation and sympathetic deactivation. Sympathetic deactivation is beneficial in the conditions of systolic or diastolic failure and detrimental in the hypovolemia conditions. To achieve beneficial effects, only niche indications are to be selected, which is at variance with a “one size fits all” approach. The learning curve extends from stable circulation (*delirium tremens*) to isolated ventilatory distress and then to acute cardioventilatory distress. A “start slow-go slow” approach is suggested. Neuroleptics supplement alpha-2 agonists, if needed, without benzodiazepines or propofol. Opioid-free analgesia is recommended. To avoid switching from conventional to cooperative sedation, alpha-2 agonists should be used as first-line sedatives.^(^46^)^ The management is itemized as follows: cognition (ataraxia,^(^5^)^ analgognosia^(^8^)^), nociception, circulation (passive leg raising,^(^122^)^ echocardiography,^(^119^,^120^)^ ventilation (fever control,^(^131^,^132^)^ agitation,^(^103^)^ inflammation,^(^31^,^33^,^35^,^110^,^166^)^ lung water,^(^123^)^ pH, PaCO_2_, hypoxemia). Evidence gathered from a randomized trial using a clear-cut design^(^73^,^74^)^ may extend the preliminary outcome data^(^37^-^45^)^ and implement the present suggestions.
